# Posterior condylar offset and clinical outcomes in medial pivot total knee arthroplasty: A comparison of mechanical and kinematic alignment

**DOI:** 10.1002/jeo2.70679

**Published:** 2026-03-03

**Authors:** Amir Koutp, Konstanze Huetter, Rene Schroedter, Lukas Leitner, Andreas Leithner, Patrick Sadoghi

**Affiliations:** ^1^ Department of Orthopaedics and Trauma Medical University of Graz Graz Austria; ^2^ Alps Surgery Institute Clinique Generale Annecy Annecy France; ^3^ Department of Orthopaedics and Trauma Surgery, Musculoskeletal University Center Munich (MUM) LMU University Hospital Munich Germany

**Keywords:** kinematic alignment, mechanical alignment, medial pivot, posterior condylar offset, total knee arthroplasty

## Abstract

**Purpose:**

The purpose of this study was to compare preservation of posterior condylar offset (PCO) and posterior condylar offset ratio (PCOR) after medial pivot (MP) total knee arthroplasty (TKA) performed with kinematic (KA) or mechanical alignment (MA), and to assess whether changes in these parameters associate with 2‐year clinical outcomes.

**Methods:**

This post hoc radiographic analysis of a prospective randomised trial included 200 patients (100 KA, 100 MA). PCO and PCOR were measured on standardised pre and postoperative lateral radiographs. Postoperative values were compared between groups using analysis of covariance, adjusting for preoperative measurements. Associations between changes in PCO/PCOR and 2‐year patient‐reported outcome measures (PROMs) and range of motion (ROM) were assessed in a subset of 100 patients using multivariable models.

**Results:**

Two hundred patients were included radiographically (MA 100, KA 100; mean age 68.9 ± 9.2 years). Adjusted between‐group differences were 1.23 mm for postoperative PCO (90% confidence interval [CI]: −0.10 to 2.56; *p* = 0.13) and 0.023 for postoperative PCOR (90% CI 0.003 to 0.043; *p* = 0.06). Equivalence within ± 2.0 mm (PCO) and ± 0.02 (PCOR) was not demonstrated. Change‐score analyses were concordant (ΔPCO difference 1.02 mm; 90% CI: −0.50 to 2.54; *p* = 0.27; ΔPCOR difference 0.026; 90% CI: −0.012 to 0.064; *p* = 0.26). In 100 patients with complete paired radiographic and 2‐year clinical data, ΔPCO/ΔPCOR showed no significant associations with PROMs or ROM after adjustment. Interobserver reliability was excellent (intraclass correlation coefficient [ICC] 0.93 for PCO; 0.89 for PCOR).

**Conclusion:**

In MP TKA, KA and MA did not differ significantly in preserving PCO or PCOR; however, statistical equivalence within prespecified margins was not established. Changes in PCO/PCOR were not associated with 2‑year outcomes, suggesting that precise surgical execution, including specific rotational strategies, rather than alignment philosophy alone, is key to maintaining posterior femoral geometry in this implant design.

**Level of Evidence:**

Level III, post hoc analysis of data from a randomised controlled trial.

**Abbreviations**
ANCOVA: analysis of covariance
BMI: body mass index
CI: bonfidence interval
FJS: forgotten joint score
ICC: intraclass correlation coefficient
KA: kinematic alignment
KSS: Knee Society Score
MA: mechanical alignment
MP: medial pivot
OKS: Oxford Knee Score
PCO: posterior condylar offset
PCOR: posterior condylar offset ratio
PROMs: patient‐reported outcome measures
ROM: range of motion
SD: standard deviation
STROBE: strengthening the reporting of observational studies in epidemiology
TKA: total knee arthroplasty
TOST: two one‐sided tests
WOMAC: Western Ontario and McMaster Universities Osteoarthritis Index

## INTRODUCTION

Accurate component positioning and restoration of physiological knee mechanics are central aims of total knee arthroplasty (TKA) [1, 20, 21, 26]. Mechanical alignment (MA) has traditionally been the reference standard, targeting a neutral mechanical axis through standardised bone resections and implant positioning [14, 23]. MA TKA prioritises creating a rectangular, balanced flexion‐extension gap, often requiring soft‐tissue releases to achieve this target. While effective for coronal alignment and implant durability, MA may alter the native joint line orientation and modify knee kinematics in some patients [30].

Kinematic alignment (KA) has emerged as an alternative that seeks to restore the patient's prearthritic anatomy including femoral condylar geometry, joint line inclination and soft‐tissue balance by aligning components to patient‐specific joint axes to better replicate native kinematics [9, 14, 18, 29]. In contrast to MA, unrestricted KA aims to resurface the native joint lines and accepts the resulting ligamentous laxity profile without planned releases, seeking to restore the patient's prearthritic soft‐tissue envelope [13].

Posterior condylar offset (PCO) [5] and the posterior condylar offset ratio (PCOR) describe the posterior projection of the femoral condyles relative to the femoral shaft [15]. These parameters influence flexion mechanics, femoral rollback and soft‐tissue tensioning, and their preservation has been associated with improved range of motion (ROM), particularly in deep flexion, and with more natural knee function [5, 7, 24]. Although KA is often assumed to better maintain PCO/PCOR than MA, evidence is inconclusive; implant design, posterior tibial slope and surgical technique may be equally influential [3, 17, 22].

Medial pivot (MP) designs add further nuance. By reproducing a ball‐and‐socket medial articulation, MP prostheses stabilise mid‐flexion and guide lateral rollback, potentially reducing dependence on PCO magnitude for functional flexion [2, 10, 11, 25]. Whether alignment philosophy meaningfully affects PCO/PCOR preservation in MP TKA remains uncertain.

The aim of this study was to compare preservation of PCO and PCOR after MP TKA performed with MA or KA, and to examine their associations with 2‐year clinical outcomes. It was hypothesised that KA would result in smaller changes in PCO and PCOR than MA, reflecting closer restoration of native femoral anatomy.

## METHODS

### Study design and setting

This was a post hoc radiographic analysis of an ongoing prospective randomised study of TKA performed with MA or KA registered at ClinicalTrials.gov (Identifier: NCT04436211). This study was conducted and reported in accordance with the strengthening the reporting of observational studies in epidemiology (STROBE) guidelines. The analysis set comprised the first 200 consecutively enrolled patients treated at a single high‐volume arthroplasty centre between October 2020 and December 2022. Institutional approval for this analysis of anonymized trial data was obtained.

### Participants

The radiographic analysis set comprised 200 primary TKAs for end‐stage osteoarthritis (MA 100, KA 100), all implanted with the same modern generation MP design (GMK Sphere, Medacta International) by one senior surgeon using a standardised peri‐operative protocol. The clinical correlation subset (*n* = 100; MA 50, KA 50) consisted of the first 100 enrolled patients from this cohort who had previously reached the 2‐year time point and for whom clinical outcomes were available. These clinical data were previously obtained for a separate analysis [19].

### Eligibility criteria

Inclusion criteria were participation in the original trial and availability of pre and postoperative lateral radiographs suitable for PCO/PCOR measurement. Exclusion criteria were prior surgery on the index knee (osteotomy, fracture fixation), inflammatory arthritis, valgus deformity > 15°, fixed flexion contracture > 20°, revision TKA or radiographs failing quality criteria. The cohort predominantly consisted of varus osteoarthritis, which is typical for TKA populations; subgroup analysis of the small number of valgus knees was not performed.

### Surgical technique

All procedures were performed by the senior surgeon following the manufacturer's surgical technique, adapted for the specific alignment philosophy. The MA technique aimed for a neutral mechanical axis. Femoral resection was performed using a posterior referencing system. Crucially, femoral component rotation was set at 3° of external rotation relative to the posterior condylar axis (PCA) to achieve a rectangular flexion gap. The distal femur was resected perpendicular to the mechanical axis. The tibial cut was made perpendicular to the mechanical axis in the coronal plane with a 3° posterior slope. The goal was to achieve a balanced flexion‐extension gap; soft‐tissue releases were performed as required to achieve balance (≤2 mm medial and lateral laxity).

The KA technique aimed to restore the patient's native joint lines. The distal femoral resection was set parallel to the native distal femoral joint line, and the posterior femoral resection was set parallel to the native posterior femoral joint line, both adjusted for cartilage wear. Femoral component rotation was set at 0° relative to the native PCA, effectively resurfacing the posterior condyles. The tibial resection was performed parallel to the native tibial joint line in the coronal plane, also adjusted for wear, while maintaining the native posterior slope. Soft tissue releases were not performed as part of the KA technique.

### Radiographic acquisition and measurement

Lateral radiographs were acquired with the knee flexed 20°–30°and the beam centred at the femoral epicondyles. Standard anterior‐posterior and long‐leg standing radiographs were acquired to verify coronal alignment targets (hip‐knee‐ankle axis) as part of the primary trial protocol, but were not used for the specific PCO analysis in this study. Images were screened for lateral quality; studies with posterior condylar overlap discrepancy >2 mm or patellar malalignment > one third of patellar width were predefined for exclusion. No radiographs were excluded based on these criteria.

**Figure 1:**
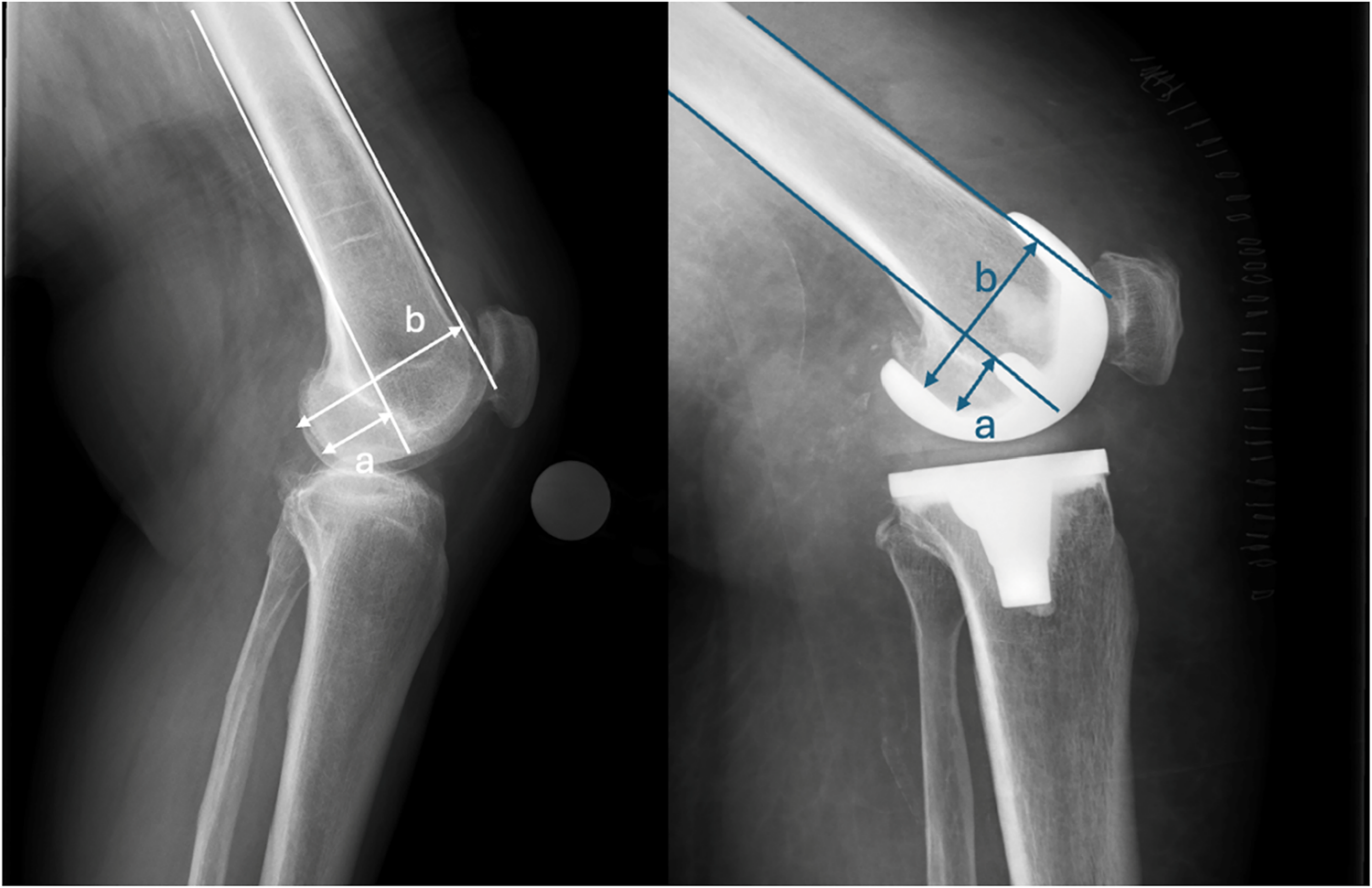
Measurement of posterior condylar offset (PCO) and posterior condylar offset ratio (PCOR) on true lateral radiographs. *a* = distance (mm) from a line tangent to the posterior femoral diaphyseal cortex to the posterior condylar margin; *b* = anteroposterior diameter (mm) of the distal femur at the same level; PCOR = *a*/*b*.

PCO was defined as the maximum perpendicular distance from a line tangent to the posterior femoral diaphyseal cortex to the most posterior point of the femoral condyles. PCOR was defined as PCO divided by the anteroposterior diameter of the distal femur at the same level. Measurement definitions followed validated methods; a schematic is provided in Figure 1.

### Observer reliability

Two observers, blinded to alignment group and outcomes, measured all images independently. Interobserver reliability was quantified using two‐way random‐effects intraclass correlation coefficients (ICCs) (absolute agreement, single measures) with 95% confidence intervals.

### Variables and outcomes

The primary endpoint was postoperative PCO (and PCOR) adjusted for baseline values. Secondary endpoints were change in PCO (ΔPCO) and change in PCOR (ΔPCOR) from pre to postoperative radiographs. Prespecified clinical secondary analyses examined associations of ΔPCO/ΔPCOR with 2‐year patient‐reported outcome measures (PROMs) (Oxford Knee Score [OKS], Western Ontario and McMaster Universities Osteoarthritis Index [WOMAC], forgotten joint score [FJS], Knee Society Score [KSS]) and ROM.

### Statistical analysis

The primary between‐group comparison used analysis of covariance (ANCOVA) with postoperative PCO (or PCOR) as the dependent variable, preoperative value as covariate and alignment group (MA vs. KA) as the fixed factor. For clinical outcomes, multivariable linear regression models were fitted with the clinical score as the dependent variable and either ΔPCO or ΔPCOR as the predictor, adjusting for age, sex, BMI, preoperative score (where available) and alignment group. Model assumptions (linearity, normality of residuals and homoscedasticity) were verified; variance inflation factors <1.3 indicated acceptable collinearity. Pearson correlations were prespecified as supportive analyses. To account for multiplicity across PROMs, a false discovery rate (Benjamini–Hochberg) of *q* = 0.05 was applied separately to the ΔPCO and ΔPCOR predictor families. Effect sizes with 95% confidence intervals are reported; two‐sided *α* = 0.05.

Secondary equivalence testing used two one‐sided tests (TOST) with prespecified bounds of ± 2.0 mm for ΔPCO and ± 0.02 for ΔPCOR. Equivalence was concluded if the 90% confidence interval of the group difference lay entirely within these bounds, and both one‐sided tests were significant at *α* = 0.05. Analyses were performed in R version 4.4.1 (R Foundation for Statistical Computing) using base stats, emmeans and TOSTER packages.

### Sample size and precision

With 100 patients per group, the detectable between‐group difference at 80% power (*α* = 0.05) was approximately 2.59 mm for ΔPCO (observed SD 6.54 mm) and 0.065 for ΔPCOR (observed SD: 0.164). For clinical correlations (*n* = 100), the study had 80% power to detect |*r* | ≥ 0.28. These estimates contextualise precision and do not alter the prespecified analysis plan.

### Ethics

Approved by the Ethics Committee of the Medical University of Graz (31‐176 ex 18/19); informed consent was obtained from all participants. The present study was performed in accordance with the Declaration of Helsinki. The present secondary analysis of anonymized data was approved with consent waived.

## RESULTS

### Study population

**Figure 2:**
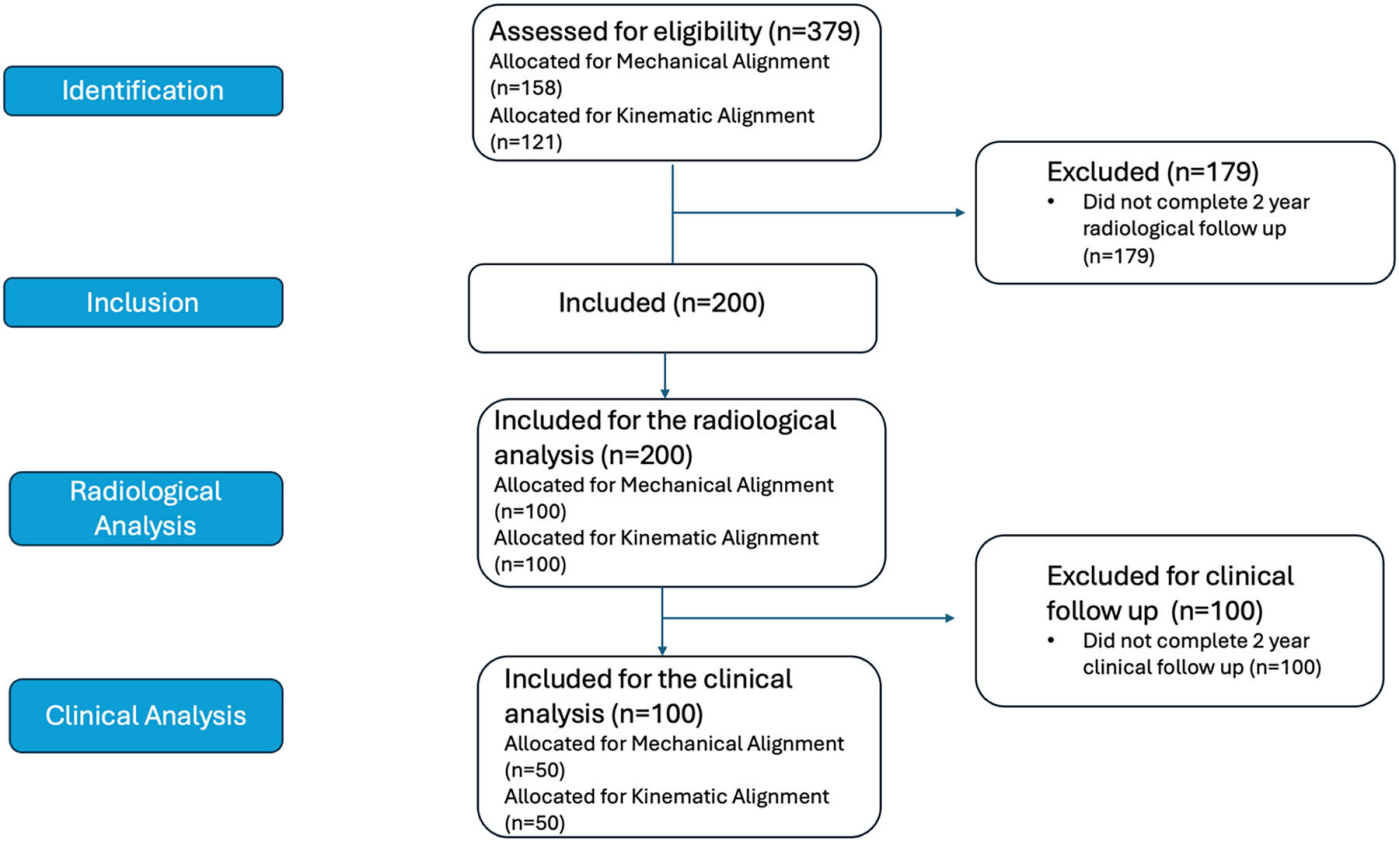
STROBE‐compliant flowchart of patient inclusion. The chart details the number of patients assessed for eligibility in the parent RCT, those randomised to the MA (*n* = 100) or KA (*n* = 100) groups, those included in the final radiographic analysis (*n* = 200), and the subset of patients with complete 2‐year clinical follow‐up included in the clinical correlation analysis (*n* = 100; MA = 50, KA = 50). KA, kinematic alignment; MA, mechanical alignment; RCT, randomized controlled trial; STROBE, strengthening the reporting of observational studies in epidemiology.

The radiographic analysis included 200 patients (MA 100, KA 100). The clinical correlation subset comprised the first 100 of these patients to reach 2‐year follow‐up (MA 50, KA 50), for whom complete paired radiographic and clinical data were available (Figure 2). The cohort mean age was 68.9 ± 9.2 years. Interobserver reliability was excellent (PCO ICC 0.93; PCOR ICC 0.89).

### Primary between‐group comparison (ANCOVA)

**Figure 3:**
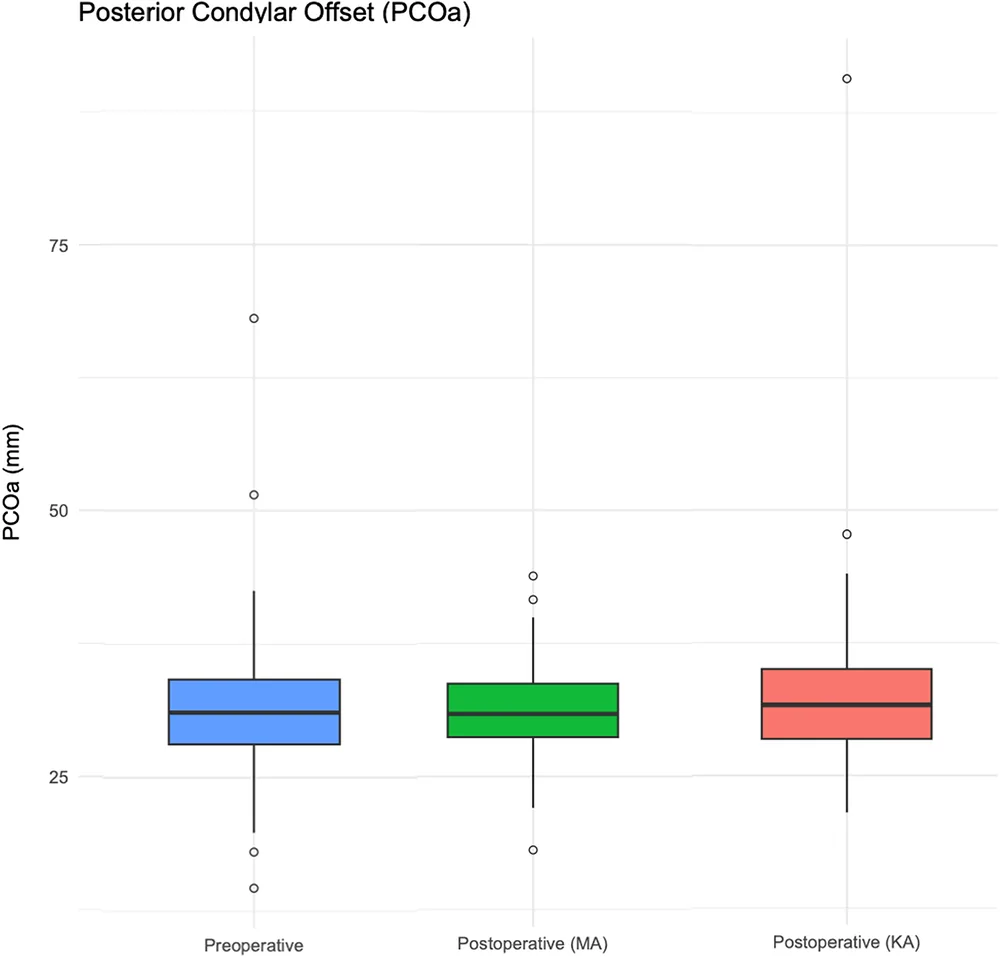
Boxplot of posterior condylar offset (PCO) measurements across groups. Preoperative values are shown for the entire cohort (*n* = 200), while postoperative values are separated by alignment strategy: mechanical alignment (MA, *n* = 100) and kinematic alignment (KA, *n* = 100). Boxes represent the interquartile range (IQR), with horizontal lines indicating the median. Whiskers extend to 1.5 × IQR; outliers are shown as individual points.

Adjusted for preoperative values, the KA–MA difference in postoperative PCO was 1.23 mm (90% CI: − 0.10 to 2.56; *p* = 0.13), and in postoperative PCOR was 0.023 (90% CI: 0.003 to 0.043; *p* = 0.06). Thus, no statistically significant between‐group differences were detected. The distributions of pre and postoperative measurements are shown in Figure 3.

### Supportive change‐score analyses

Within‐group changes were small and nonsignificant (MA ΔPCO: −0.11 mm, *p* = 0.86; KA ΔPCO + 0.92 mm, *p* = 0.21; MA ΔPCOR + 0.0056, *p* = 0.79; KA ΔPCOR − 0.0203, *p* = 0.051). The between‐group differences in ΔPCO and ΔPCOR were not statistically significant (both *p* > 0.25).

### Equivalence testing

Secondary equivalence analyses did not demonstrate equivalence within the prespecified margins. For ΔPCO, the KA–MA mean difference was 1.02 mm (90% CI: −0.50 to 2.54), exceeding the ± 2.0 mm bounds; equivalence was not established. For ΔPCOR, the difference was 0.026 (90% CI: −0.012 to 0.064), exceeding the ± 0.02 bounds; equivalence was not established. ANCOVA‐adjusted comparisons yielded similar conclusions (postoperative PCO 1.23 mm, 90% CI: −0.10 to 2.56; postoperative PCOR 0.023, 90% CI: 0.003 to 0.043).

### Clinical correlations

**Figure 4:**
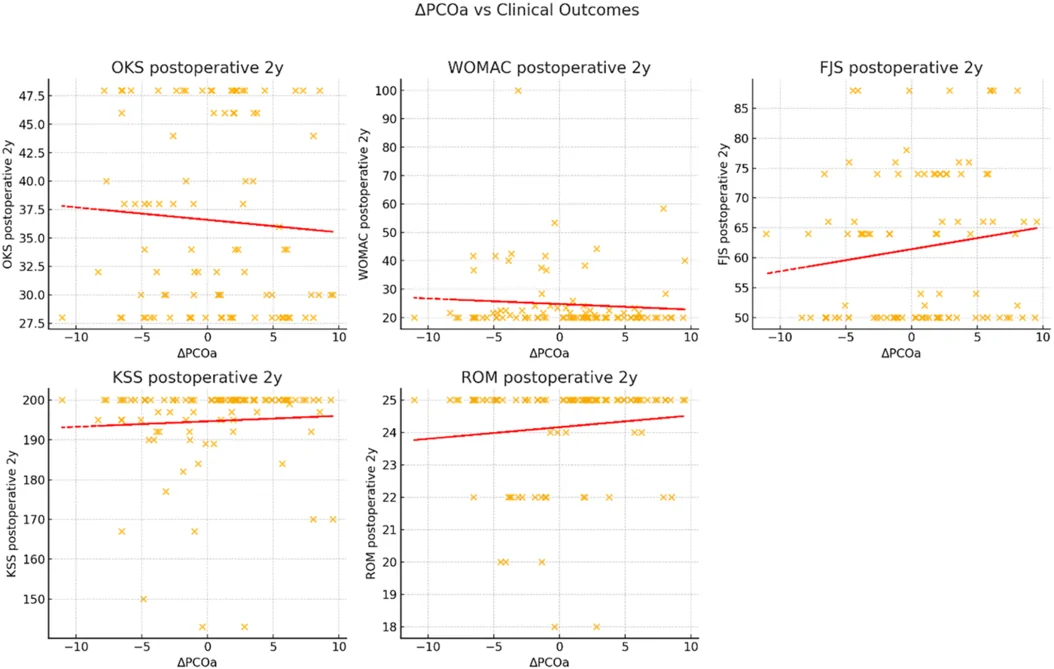
Scatter plots showing the relationship between the change in posterior condylar offset (ΔPCO) from preoperative to 2‐year postoperative radiographs and key clinical outcomes at 2 years: Oxford Knee Score (OKS), Western Ontario and McMaster Universities Osteoarthritis Index (WOMAC), Forgotten Joint Score (FJS), total Knee Society Score (KSS) and range of motion (ROM). Each plot includes a simple linear regression line (red, dashed). No significant correlations were observed.

**Figure 5:**
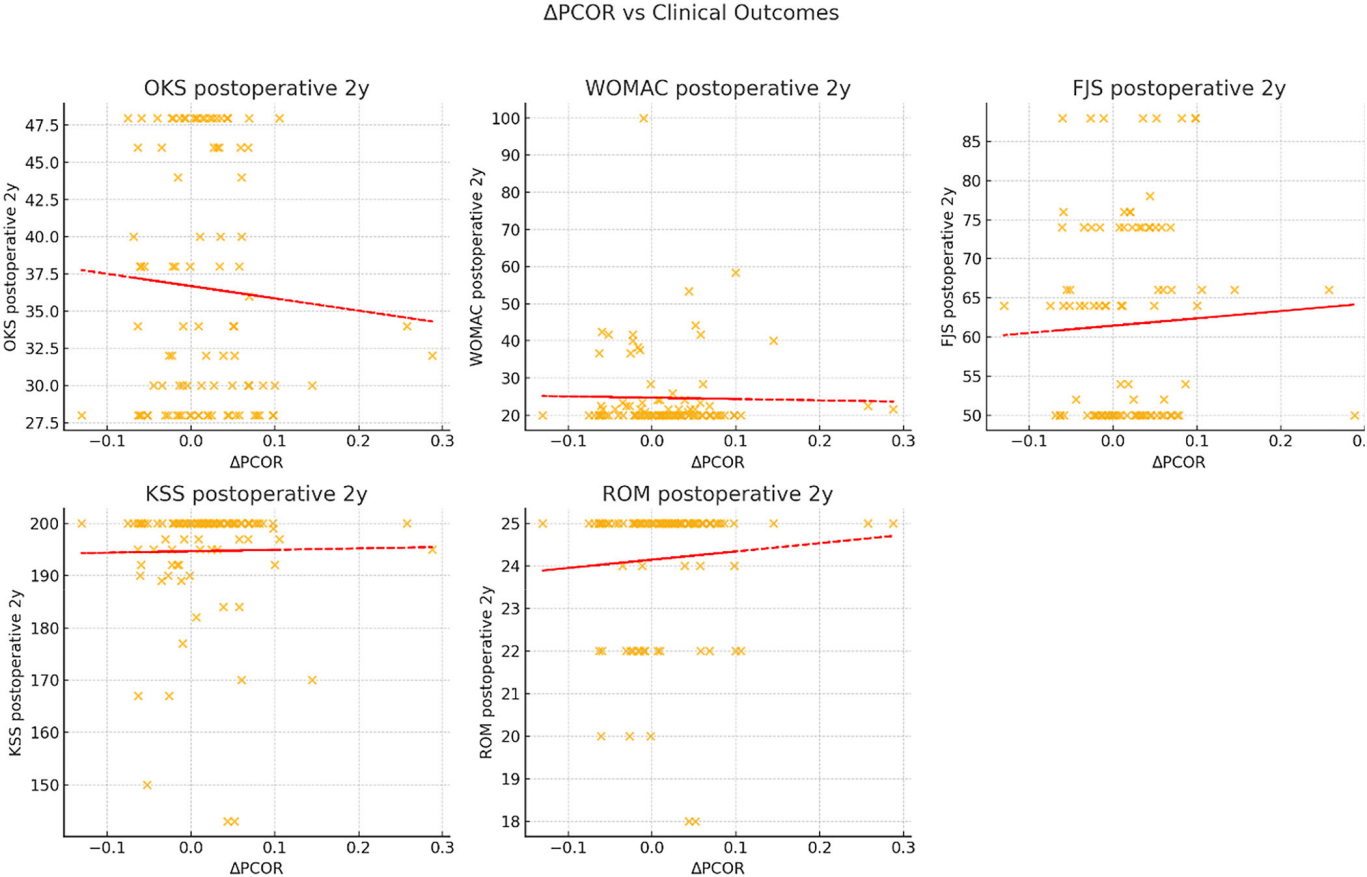
Scatter plots showing the relationship between the change in posterior condylar offset ratio (ΔPCOR) from preoperative to 2‐year postoperative radiographs and key clinical outcomes at 2 years: Oxford Knee Score (OKS), Western Ontario and McMaster Universities Osteoarthritis Index (WOMAC), Forgotten Joint Score (FJS), total Knee Society Score (KSS) and range of motion (ROM). Each plot includes a simple linear regression line (red, dashed). No significant correlations were observed.

In the subset of 100 patients (MA 50, KA 50) with complete paired radiographic and 2‐year clinical data, adjusted multivariable regression (*n* = 100) showed no significant associations between ΔPCO or ΔPCOR and 2‑year OKS, WOMAC, FJS, KSS or ROM after multiplicity control; all effect sizes were small, and 95% CIs crossed the null (Table 1). For example, each 1‑mm increase in ΔPCO was associated with −0.11 points in OKS (95% CI: − 0.48 to 0.26; *p* = 0.55; FDR‑adjusted *p* = 0.55). Scatter plots of bivariate relationships are provided in Figures 4 and 5.

**Table 1:** Adjusted multivariable regression of ΔPCO and ΔPCOR with 2‐year clinical outcomes (OKS, WOMAC, FJS, KSS and ROM).

Outcome	Predictor	*β*	95% CI	*p*	pFDR	*n*
FJS (2 years)	ΔPCO	0.39	−0.13 to 0.90	0.141	0.42	100
ROM (2 years)	ΔPCO	0.04	−0.03 to 0.11	0.252	0.42	100
KSS (2 years)	ΔPCO	0.17	−0.33 to 0.67	0.496	0.546	100
OKS (2 years)	ΔPCO	−0.11	−0.48 to 0.26	0.546	0.546	100
WOMAC (2 years)	ΔPCO	−0.28	−0.77 to 0.20	0.249	0.42	100
FJS (2 years)	ΔPCOR	11.04	−28.74 to 50.83	0.583	0.688	100
ROM (2 years)	ΔPCOR	3.03	−2.30 to 8.37	0.262	0.688	100
KSS (2 years)	ΔPCOR	9.19	−29.29 to 47.68	0.636	0.688	100
OKS (2 years)	ΔPCOR	−5.65	−33.53 to 22.23	0.688	0.688	100
WOMAC (2 years)	ΔPCOR	−9.21	−46.38 to 27.96	0.624	0.688	100

*Note*: Models adjusted for age, sex, BMI, preoperative score (where available) and alignment group (KA vs. MA). β per 1‐mm ΔPCO or per unit ΔPCOR, with 95% CI, raw p and FDR‐adjusted p (pFDR) are shown.

Abbreviations: BMI, body mass index; CI, confidence interval; FJS, Forgotten Joint Score; KA, kinematic alignment; KSS, Knee Society Score; MA, mechanical alignment; OKS, Oxford Knee Score; PCO, posterior condylar offset; PCOR, posterior condylar offset ratio; pFDR, false discovery rate–adjusted *p*‐Value; ROM, range of motion; WOMAC, Western Ontario and McMaster Universities Osteoarthritis Index.

## DISCUSSION

The most important finding of this study was that in MP total knee arthroplasties, no statistically significant differences were detected between MA and KA in the preservation of PCO or PCOR. The primary ANCOVA showed no between‐group differences in postoperative PCO/PCOR after adjustment for baseline values, and change‐score analyses were concordant. Measurement reliability was excellent (PCO ICC 0.93; PCOR ICC 0.89). Importantly, adjusted multivariable regression with false discovery rate control demonstrated no associations between ΔPCO/ΔPCOR and 2‐year outcomes (OKS, WOMAC, FJS, KSS, ROM). Taken together, these findings suggest that, within the narrow range of geometric variation observed, preservation of posterior condylar anatomy is unlikely to be a determining factor for short‐ to mid‐term function in MP TKA, when performed with these meticulous techniques. This minimal difference between groups is further explained by the surgical technique itself; the standard application of 3° external rotation in the MA group is a deliberate manoeuvre that preserves posteromedial bone, thus minimising the alteration to PCO compared to a purely theoretical mechanical cut and bringing the geometric result closer to that of KA.

Secondary equivalence testing provided a complementary perspective. Although no statistically significant differences in ΔPCO or ΔPCOR were observed, the 90% confidence intervals for the KA–MA differences extended beyond the prespecified equivalence margins (± 2.0 mm for ΔPCO; ± 0.02 for ΔPCOR). Equivalence was therefore not demonstrated. Collectively, these results indicate that while large differences are unlikely, small between‐group differences cannot be excluded by the present study.

Our findings are consistent with contemporary evidence that, in modern implant designs, particularly MP prostheses, small variations in PCO have limited clinical impact [27]. Earlier work, largely in cruciate‐retaining designs, emphasised that reductions in PCO exceeding approximately 3 mm can impair postoperative flexion by altering femoral rollback [12, 22, 31, 32]. However, more recent studies in posterior‐stabilised and MP designs report that modest changes in PCO rarely translate into functional deficits [4, 6, 8, 27].

The MP design is biomechanically distinct from conventional posterior‐stabilised TKA. By creating a highly congruent, ball‐in‐socket articulation medially, the MP implant provides a stable pivot point throughout flexion. This intrinsic stability, guided by the articular geometry itself rather than solely by ligamentous tension acting on the posterior condyles, encourages physiological lateral rollback and may reduce the system's dependence on the precise magnitude of PCO for achieving deep, stable flexion [2, 10, 11, 16, 25, 28]. This intrinsic stability may reduce dependence on PCO magnitude for achieving deep flexion, as rollback is guided more by articular geometry and less by posterior condylar anatomy. Furthermore, in MP designs, load transfer and kinematics may remain optimised despite minor variations in condylar position, which could explain the absence of clinically meaningful correlations in our cohort [2, 10, 11].

In the present study, mean changes in both ΔPCO and ΔPCOR were well within the thresholds proposed by Zhong et al. [32], who identified an optimal PCO variation within 2.85 mm as associated with improved WOMAC and FJS scores. Variations smaller than this, as seen here, are unlikely to produce functional deficits. Similarly, a meta‐analysis by Zhang et al. [31] found that deviations below 2–3 mm generally have no adverse impact on outcomes, supporting the notion that the small geometric changes observed in our MA and KA groups are clinically irrelevant. The comparable performance of both alignment strategies suggests that in MP TKA, surgical precision in alignment—including deliberate rotational adjustments in MA—can preserve femoral geometry equally well without compromising patient function.

### Clinical relevance

For surgeons using MP designs, these data indicate that alignment philosophy alone (KA vs. MA) is unlikely to materially influence PCO/PCOR or 2‑year outcomes when surgery is executed precisely. Pragmatically, attention should focus on avoiding large PCO reductions, which have been associated in other studies with poorer outcomes (e.g., >3 mm) [12, 22, 31, 32], through appropriate femoral sizing and anteroposterior positioning, with routine postoperative lateral radiographs used to confirm that substantial PCO loss has not occurred.

### Limitations

This study has limitations. It is a post hoc analysis of a prospective randomised study, with potential selection bias, particularly for the clinical correlation subset (*n* = 100 complete pairs). The cohort exhibited a relatively narrow range of ΔPCO/ΔPCOR, limiting inference about larger deviations. Additionally, while no associations were found between PCO changes and clinical scores, we must acknowledge that the PROMs used (OKS, FJS, WOMAC) may lack the sensitivity to detect subtle kinematic changes, and the variations in scores observed were below the minimal clinically important difference (MCID) for these instruments.

Second, the surgical technique for MA employed a fixed 3° external rotation relative to the PCA. We acknowledge that MA practices vary, with some surgeons utilising gap‐balancing techniques that may result in variable femoral rotation. Therefore, our findings may not be generalisable to all MA techniques, particularly those that do not use posterior referencing. Third, PCO/PCOR were measured on 2D lateral radiographs, which have an inherent measurement error of up to 2 mm and are sensitive to knee flexion and rotation. While we used standardised acquisition protocols, predefined quality thresholds, and demonstrated excellent interobserver reliability (ICC > 0.89), this context reinforces our conclusion that the small, statistically nonsignificant differences observed are unlikely to be clinically meaningful, as they fall within the range of potential measurement error. Although adjusted models accounted for age, sex, BMI, baseline score and alignment group, unmeasured confounding (e.g., posterior tibial slope, component size/rotation) cannot be excluded. The study was adequately powered to exclude large differences but was underpowered for narrow equivalence margins; small between‐group differences below ≈ 2.6 mm (ΔPCO) or ≈ 0.065 (ΔPCOR) may have gone undetected. Finally, the cohort predominantly consisted of varus knees; thus, conclusions regarding specific strategies for severe valgus deformities cannot be drawn from this dataset.

## CONCLUSION

In MP TKA, KA and MA showed no statistically significant differences in postoperative PCO or PCOR after adjustment for baseline values. Changes in PCO/PCOR were not associated with 2‑year PROMs or ROM. These findings indicate that, for modern MP designs, precise surgical execution, including specific rotational strategies, rather than alignment philosophy alone, is central to maintaining posterior femoral geometry and achieving comparable short‐ to mid‐term outcomes. Furthermore, the intrinsic stability of the MP design may render it more accommodating to minor geometric variations than historical designs, resulting in comparable short‐ to mid‐term clinical outcomes regardless of the alignment strategy employed.

## AUTHOR CONTRIBUTIONS

**Amir Koutp**: Study design; data collection; statistical analysis; manuscript writing. **Lukas Leitner**: Data analysis; statistical consultation; manuscript editing. **Rene Schroedter**: Data acquisition; radiographic analysis; manuscript editing. **Konstanze Huetter**: Data analysis; interpretation; manuscript review. **Andreas Leithner**: Conceptualisation; critical manuscript revision. **Patrick Sadoghi**: Principal investigator; study conception; supervision; final manuscript approval.

## CONFLICT OF INTEREST STATEMENT

Andreas Leithner: Industry grants from DePuy Synthes, Johnson & Johnson, alphamed and Medacta. Patrick Sadoghi receives Industry grants from DePuy Synthes, Johnson & Johnson, alphamed and Medacta; Editorial Board Member for JOA, KSSTA and Arthroscopy. The remaining authors declare no conflicts of interest.

## ETHICS STATEMENT

Approved by the Ethics Committee of the Medical University of Graz (31‐176 ex 18/19). All patients provided informed consent.

## Data Availability

Data are available from the corresponding author upon reasonable request.
